# Time-Dependent Changes in the Biofluid Levels of Neural Injury Markers in Severe Traumatic Brain Injury Patients–Cerebrospinal Fluid and Cerebral Microdialysates: A Longitudinal Prospective Pilot Study

**DOI:** 10.1089/neur.2022.0076

**Published:** 2023-03-01

**Authors:** I-Hsuan Lin, Alaa Kamnaksh, Roxanne Aniceto, Jesse McCullough, Ramsey Bekdash, Michael Eklund, Per Hamid Ghatan, Mårten Risling, Mikael Svensson, Bo-Michael Bellander, David W. Nelson, Eric Peter Thelin, Denes V. Agoston

**Affiliations:** ^1^ Department of Anatomy, Physiology and Genetics, Uniformed Services University, Bethesda, Maryland, USA.; ^2^ Department of Neuroscience, Uppsala University Hospital, Uppsala, Sweden.; ^3^ Department of Neuroscience, Department of Physiology and Pharmacology, Karolinska Institutet, Stockholm, Sweden.; ^4^ Department of Clinical Neuroscience, Department of Physiology and Pharmacology, Karolinska Institutet, Stockholm, Sweden.; ^5^ Department of Neurosurgery, Karolinska University Hospital, Stockholm, Sweden.; ^6^ Department of Perioperative Medicine and Intensive Care, Karolinska University Hospital, Stockholm, Sweden.; ^7^ Section of Perioperative Medicine and Intensive Care, Department of Physiology and Pharmacology, Karolinska Institutet, Stockholm, Sweden.; ^8^ Department of Neurology, Karolinska University Hospital, Stockholm, Sweden.

**Keywords:** biomarker, cMD, CSF, protein, temporal, traumatic brain injury

## Abstract

Monitoring protein biomarker levels in the cerebrospinal fluid (CSF) can help assess injury severity and outcome after traumatic brain injury (TBI). Determining injury-induced changes in the proteome of brain extracellular fluid (bECF) can more closely reflect changes in the brain parenchyma, but bECF is not routinely available. The aim of this pilot study was to compare time-dependent changes of S100 calcium-binding protein B (S100B), neuron-specific enolase (NSE), total Tau, and phosphorylated Tau (p-Tau) levels in matching CSF and bECF samples collected at 1, 3, and 5 days post-injury from severe TBI patients (*n* = 7; GCS 3–8) using microcapillary-based western analysis. We found that time-dependent changes in CSF and bECF levels were most pronounced for S100B and NSE, but there was substantial patient-to-patient variability. Importantly, the temporal pattern of biomarker changes in CSF and bECF samples showed similar trends. We also detected two different immunoreactive forms of S100B in both CSF and bECF samples, but the contribution of the different immunoreactive forms to total immunoreactivity varied from patient to patient and time point to time point. Our study is limited, but it illustrates the value of both quantitative and qualitative analysis of protein biomarkers and the importance of serial sampling for biofluid analysis after severe TBI.

## Introduction

Cerebrospinal fluid (CSF) is uniquely qualified for protein biomarker analysis after traumatic brain Injury (TBI) because of its proximity to the brain (for reviews, see previous works^1–3^). Cerebral microdialysis (cMD)^4^ has greatly contributed to the better understanding of changes in intracranial metabolism after TBI by enabling continuous sampling and analysis of brain extracellular fluid (bECF) for changes in lactate, pyruvate, and glucose levels.^5–9,10^ The bECF proteome can closely reflect cerebral tissue-level changes—at least at the sampling site^11^—and elevated lactate-to-pyruvate ratio was found to be associated with a specific bECF proteome consisting of cytoarchitectural and mitochondrial proteins as well as a unique peptide with a mass/charge 4733.5, a candidate protein marker of metabolic crisis in TBI patients.^12^ Additional analyses have identified changes in the bECF proteome after both experimental and clinical TBI.^13^ Studies have shown that high initial Tau levels are indicative of poor outcome,^14^ and that elevated total Tau and beta-amyloid levels correlate with injury severity in focal and/or mixed types of TBI^14,15^ and identified the inflammatory response after TBI.^16^ However, cMD is not widely performed and the qualitative, quantitative, and temporal relationships between protein biomarker levels in the CSF versus bECF thus are currently poorly understood.^1,2^

The protein biomarkers S100 calcium-binding protein B (S100B), neuron-specific enolase (NSE), Tau, and phosphorylated Tau (p-Tau) have been extensively studied in TBI,^17,18^ and their elevated serum and CSF levels have been shown to indicate the extent of neuronal, glial, and axonal damage as well as correlate with injury severity and outcome.^19–24^ In this pilot study, we used microcapillary electrophoresis-coupled western analysis (WES) to determine the qualitative, quantitative, and temporal relationships between CSF and bECF levels of S100B, NSE, Tau, and p-Tau. WES is a highly sensitive proteomic platform that requires very low sample volume (microliters), and, like traditional westerns, it can separate immunoreactive proteins by molecular weight.

## Methods

### Patients and clinical parameters

Patients in our study were a subset of a larger population from a prospective observational study performed at the North Carolina Central University at Karolinska University Hospital (Stockholm, Sweden) under ethical approval #2009/1112-31/3 by Stockholm County branch of the Central Ethical Review Board, now called the Swedish Ethical Review Authority (Table 1). Study details, including inclusion and exclusion criteria, patient management, and sample acquisition, are as described in detail earlier.^25^ For this study, we selected patients who had matching CSF and bECF samples at three acute post-injury time points (days 1, 3, and 5 as detailed in Table 2).

**Table 1.** Patient Demographics of the Study Cohort

Patient ID	6	7	10	17	11	13	14
Sex	M	M	M	M	M	M	M
Age, years	22	23	25	36	42	59	62
GCS	7	8	8	7	3	7	3
ISS	29	16	25	26	38	25	25
AIS	4	4	5	4	5	5	5
Pupil responsiveness	1	0	0	0	1	1	0
Outcome (GOS)^a^	3	4	5	4	3	3	1

^a^ Outcome was determined 6 months after the injury by a neurorehabilitation board-certified physician (P.H.G.); GOS categories: 1) dead, 2) persistent vegetative state, 3) severe disability, 4) moderate disability, and 5) low disability.

M, male; GCS, Glasgow Coma Scale; ISS, Injury Severity Score; AIS, Abbreviated Injury Scale; GOS, Glasgow Outcome Scale.

**Table 2.** An Overview of Samples Analyzed by WES

Time points	Day 1	Day 3	Day 5
Original collection time points (h) to be combined	6, 12, 18, 24 h	54, 60, 66, 72 h	102, 108, 114, 120 h
Patient nos.			
6	CSF; bECF	CSF; bECF	CSF; bECF
7	CSF; bECF	CSF; bECF	CSF; bECF
10	CSF; bECF	CSF; X	CSF; X
11	CSF; bECF	CSF; bECF	CSF; bECF
13	CSF; bECF	X; X	CSF; bECF
14	CSF; bECF	CSF; bECF	X; X
17	CSF; bECF	CSF; bECF	CSF; bECF

Note: “X” refers to missing samples.

WES, western analysis; CSF, cerebrospinal fluid; bECF, brain extracellular fluid.

### Biosamples

#### Cerebrospinal fluid

CSF was collected using a catheter (conventional ventricular drain) placed in the ventricle and connected to a pump (Liquoguard^®^) collecting CSF at a rate of 2 mL/h, as long as intracranial pressure was >2 mm Hg. CSF was collected every 6 h, centrifuged, and the supernatant was transferred into collection tubes and stored in a −70°C freezer.

#### Brain extracellular fluid

cMD was performed as part of the clinical routine at the Neurointensive Care Unit of the Department of Neurosurgery at the Karolinska Hospital to monitor brain metabolism.^7–9,26–28^ A 0.6-mm-wide microdialysis catheter with a 10-mm dialysis membrane at its tip (100-kDa cutoff) was surgically introduced into the brain tissue of interest (in the border zone close to the injury). A pump perfused the interior of the catheter with a perfusion fluid, which equilibrated with the interstitial tissue surrounding the catheter. Equilibration occurred by diffusion of chemicals over the dialysis membrane. Using a perfusion flow of 0.3 μL/min, the recovery of glucose, lactate, pyruvate, and glutamate in the dialysate was ∼70% of the concentration in the interstitial fluid.^29^ Samples were continuously collected into microvials analyzed at bedside by a CMA 600 microdialysis analyzer every hour for changes in glucose, pyruvate, lactate, glycerol, and glutamate. In the same area, a similar catheter with a 100-kDa cutoff was introduced to collect proteins. The perfusion fluid was the same as for the 20-kDa catheter, but samples were collected every sixth hour and frozen at −70°C. The final collection tubes contained a protease and phosphatase inhibitor cocktail.^30,31^

Because of the low protein concentrations of bECF samples, we needed to combine four consecutive collections (e.g., 6, 12, 18, and 24 h) to be able to assay them using WES (see Table 2). To match the bECF samples, we also pooled equal volumes of CSF samples collected at time points matching the bECF collections. The final, combined bECF and CSF samples represent three post-injury time points: days 1, 3, and 5 (Table 2).

### Protein analysis

CSF and bECF samples were analyzed by using WES (Simple Western microcapillary-based Western; ProteinSimple, Santa Clara, CA). Samples were diluted with 5X Fluorescent Master Mix (400 mM of dithiothreitol and 5X Sample Buffer; Prod # SM-W004; ProteinSimple), making 0.48 mg/mL as the final protein concentration for CSF. Samples and standard ladders were denatured at 70°C for 20 min, then set on ice for 10 min. Primary antibody dilutions were optimized for CSF and bECF samples using antigen-antibody binding titration before the assay (Supplementary Table S1).

The WES performs protein separation, blocking, incubation with the primary and horseradish peroxidase (HRP)-conjugated secondary antibodies, washing steps, and signal detection automatically.^32^ Samples, along with the diluted primary antibodies, HRP-conjugated secondary antibody, detection reagents, and wash buffers, were loaded onto the Simple Western assay plates according to the company's protocol. Plates were centrifuged at 2500 rpm for 5 min at room temperature, then the 25-slot microcapillary cartridge and plates were placed in the WES platform for size selection to be completed (3 h). Immunodetection was performed using the WES's default setting for the 12- to 230-kDA size-based assay. Chemiluminescent signal intensities were acquired by using the company's Compass software. Intensities were normalized to a signal-to-noise ratio >10. Relative abundance of each protein was then calculated as the area under the curve (AUC) for each of the detected peaks .

## Results

**FIG. 1.**
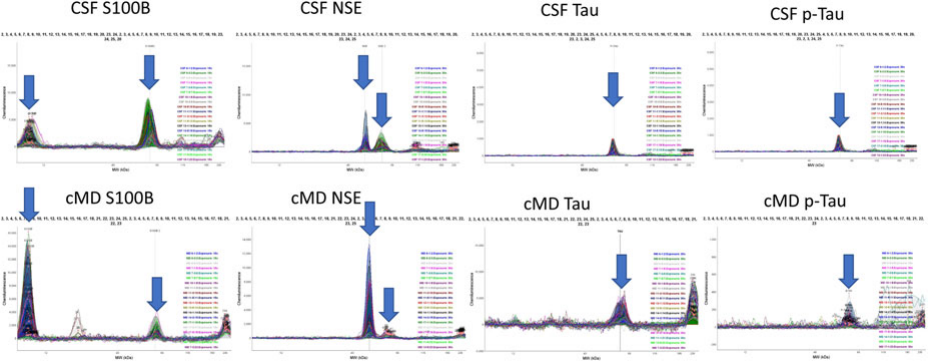
Distribution of immunoreactivities of S100B, NSE, Tau, and p-Tau in CSF and bECF samples. Blue arrows point to the immunoreactive peaks that were quantified. bECF, brain extracellular fluid; CSF, cerebrospinal fluid; NSE, neuron-specific enolase; p-Tau, phosphorylated Tau; S100B, S100 calcium-binding protein B.

In addition to the expected ∼10-kDa S100B immunoreactivity, we detected a second S100B peak at ∼60 kDa in every CSF and bECF sample, but the contribution of the two immunoreactivities differed between CSF and bECF samples (Fig. 1 and Table 3), with most patients showing CSF: 60 kDa >10 kDa vs. bECF 10 kDa >60 kDa. A few bECF samples contained a third, very small S100B immunoreactive peak at ∼20 kDa. Similarly, for NSE, in addition to the expected ∼50-kDa immunoreactive peak, there was a second immunoreactive peak at ∼60 kDa detected in all CSF samples, but it was barely detectable in bECF samples. Again, the ratio between the different immunoreactive forms varied from patient to patient (Fig 1 and Table 3). We detected the expected ∼55-kDa Tau and p-Tau immunoreactivities in CSF as well as in bECF samples, but in bECF samples there was a second Tau and p-Tau peak at >200 kDa, likely representing large Tau and p-Tau proteins likely aggregated *in vitro*.

**Table 3.** Percentage Distribution of Different Immunoreactive Forms of S100B and NSE in Different Biofluid Compartments

Marker	S100B				NSE			
Biofluid	CSF		bECF		CSF		bECF	
MW (kDa)	10 kDa	60 kDa	10 kDa	60 kDa	50 kDa	60 kDa	50 kDa	60 kDa
Patients								
PT 6	32.6	67.3	N/A	100	68.6	31.3	100	N/A
PT 7	56.3	43.6	76.1	23.8	66.9	33.01	86.7	13.2
PT 10	46.3	53.6	N/A	N/A	38.2	61.7	N/A	N/A
PT 11	12.6	87.32	63.8	36.1	21.3	78.6	55.9	44.08
PT 13	23.3	76.6	100	N/A	49.6	50.3	100	N/A
PT 14	17.1	82.8	89.6	10.3	13.2	86.7	78.8	21.1
PT 17	N/A	100	89.4	10.5	69.6	30.3	90.3	9.6

S100B, S100 calcium-binding protein B; NSE, neuron-specific enolase; CSF, cerebrospinal fluid; bECF, brain extracellular fluid; MW, molecular weight; N/A, not applicable.

**FIG. 2.**
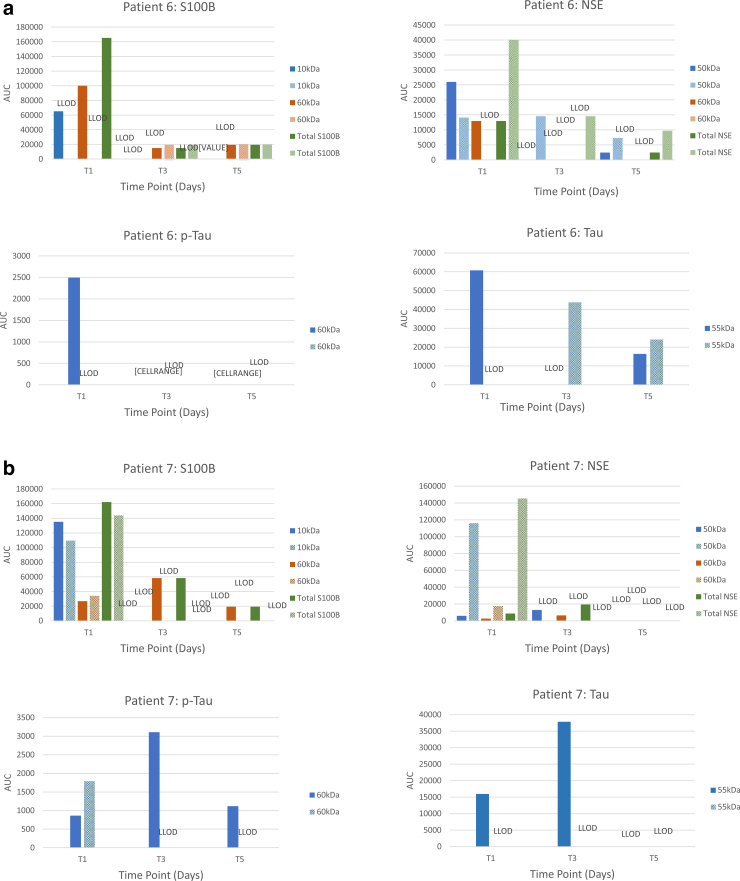

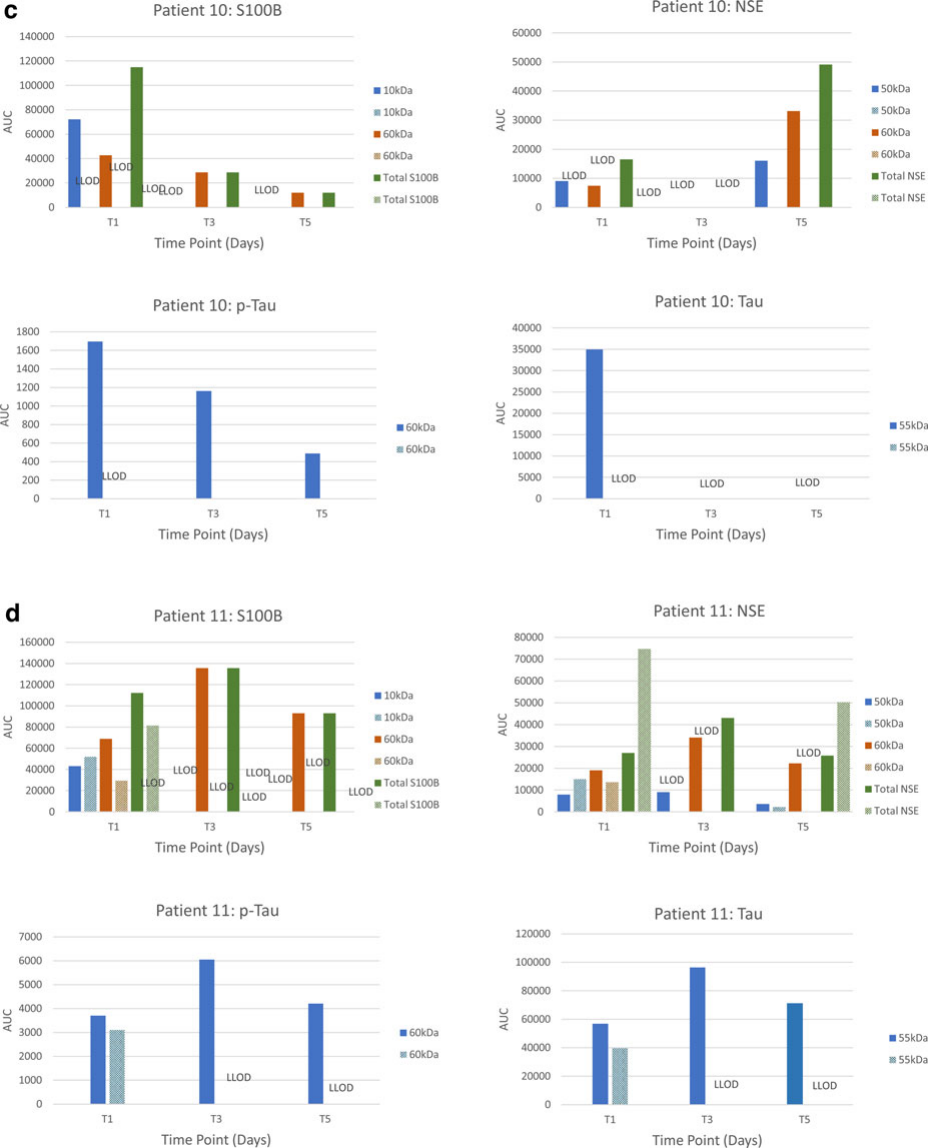

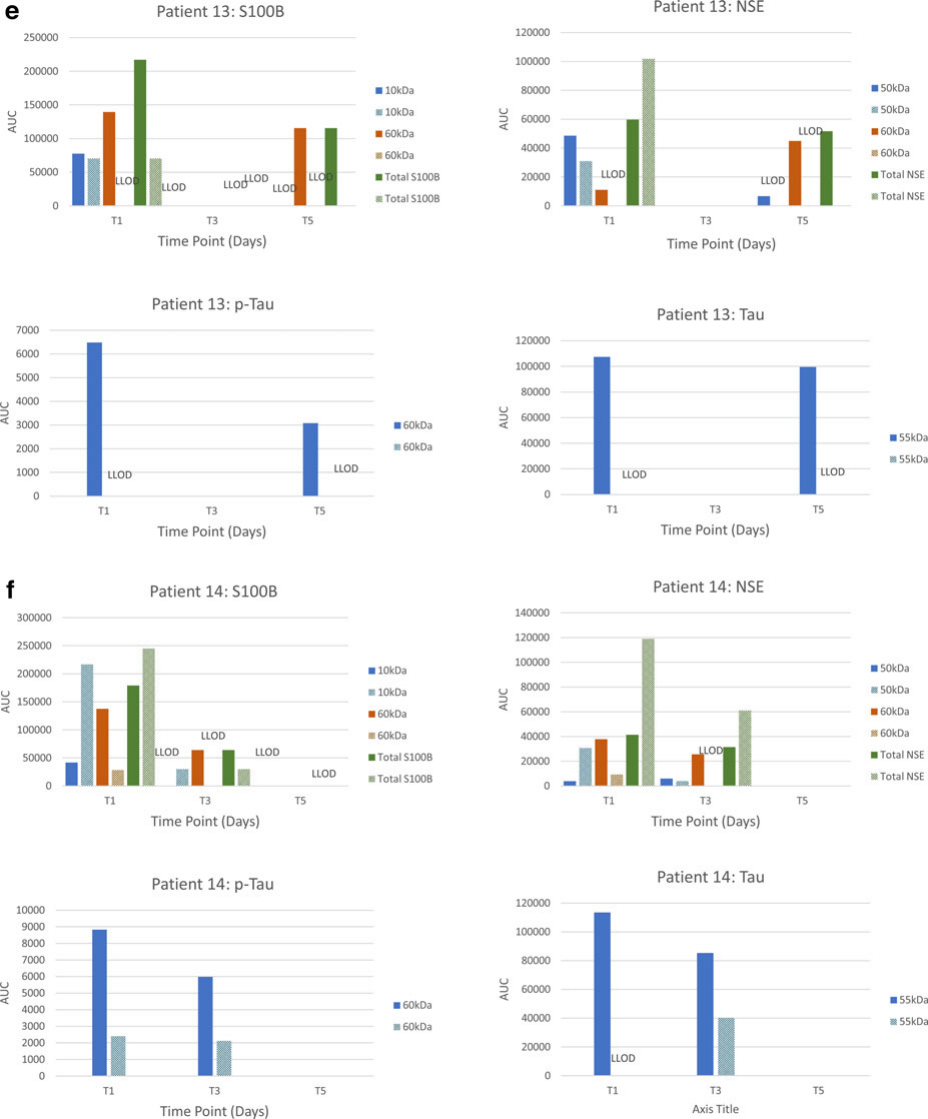

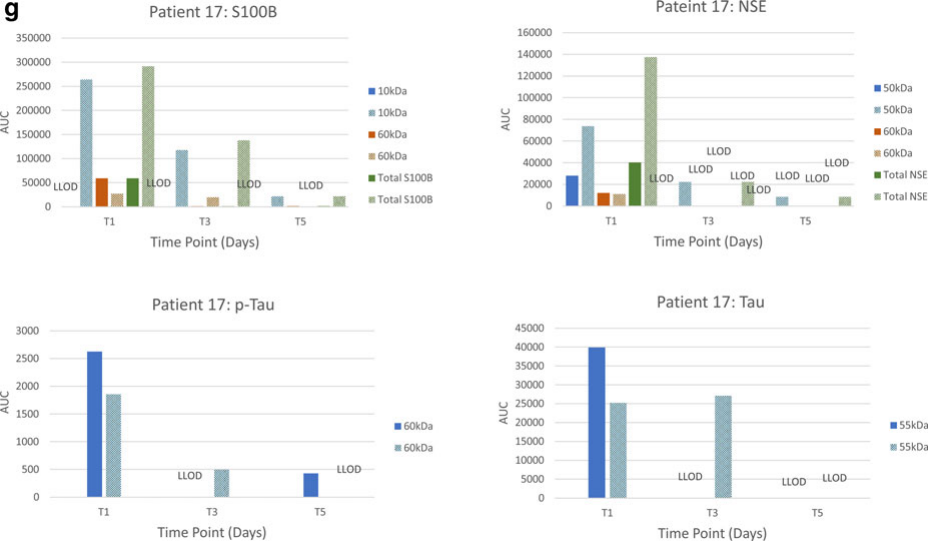
Time-dependent changes in the protein biomarker values of matching CSF and bECF samples in the individual patients (**A,** patient 6; **B,** patient 7, **C,** patient 10; **D,** patient 11; **E,** patient 13; **F,** patient 14; **G,** patient 17). Pay attention to the scales. The scales reflect the relative abundance of proteins and vary substantially between proteins and biosamples. AUC, area under the curve; bECF, brain extracellular fluid; CSF, cerebrospinal fluid; LLOD, lower limit of detection.

Semiquantitative analysis of immunoreactivities (area under the peak) showed substantial variability in biomarker levels between patients, biomarkers and their immunoreactive forms, and time points (Fig. 2A–G). But, the overall pattern of time-dependent changes showed similar trends in matching CSF and bECF samples, such that the relative concentrations of S100B and NSE immunoreactivities in both biofluids decreased over time. Highest relative concentrations of both proteins were detected at the earliest time point (T1 or day 1) and were substantially lower at T5. It should be noted that contributions of the different S100B and NSE immunoreactive forms to total immunoreactivity varied from patient to patient, time point to time point, and compartment to compartment. Tau and p-Tau levels showed similar trends, but in some patients their levels remained elevated at T5.

## Discussion

The goal of this prospective longitudinal pilot study was to understand the relationship between protein biomarker levels measured in matching CSF and bECF samples collected from severe TBI patients during the acute stage of injury. TBI-induced changes in CSF levels of protein biomarkers have been extensively studied, although most studies have used single and varying post-injury time points (for reviews, see past works^3,33^). Therefore, this is the first study that has coanalyzed matching, serially collected CSF and bECF samples.

Consistent with the earlier report that analyzed some of the same CSF samples using a different analytical platform,^25^ we found that CSF levels of S100B and NSE decreased over time. We also detected a similar temporal pattern in matching bECF samples. S100B is one of the best-characterized protein markers in TBI (for review, see past works^18,34^), and its very short half-life (∼0.5 h)^35^ makes it ideal as a marker of *de novo* release. Serum S100B levels have been established as part of the Scandinavian TBI management guidelines.^36–38^

The two biofluids CSF and bECF represent distinct intracranial environments that can differently allow and/or promote multimerization of S100B and/or secondary modifications to NSE. We detected both monomeric and multimeric (hexamer) forms of S100B protein in both CSF and bECF samples, but the ratio between the monomeric and multimeric forms varied between patients and post-injury time points. Monomeric S100B is a ∼10-kDa protein, but it forms multimers, dimers, hexamers, and even amyloids in metal ion-dependent manner.^39,40^ Extracellular S100B proteins are related to the group of proteins alarmins, also called damage-associated molecular patterns, that coordinate adaptive cellular stress response to tissue damage.^39,40^ Multimeric S100B, including hexamers, can bind to receptor for advanced glycation end product (RAGE) and Toll-like receptor-4 and activate the inflammatory response to central nervous system injury.^41–50^

Since its invention, cMD has identified changes in brain metabolism post-TBI.^4,5,9,12,14,15,51,52^ Studies have also reported injury-induced changes in the bECF proteome,^53–57^ but there have not been any studies (to our knowledge) that directly compared matching bECF and CSF samples for injury-induced changes in protein biomarker levels. It should be noted that cMD technology has known issues that can affect the outcome of protein analysis of bECF samples, such as the non-specific binding of proteins to the catheter.^52,58–60^

There are important technical issues that can be responsible for the detected S100B and NSE immunoreactive forms. The main issue, as in all antibody-based analysis, is the specificity of the antibodies. We have tested the antibodies for specificity before using them in WES, but cross-reactivity can still occur.^32,61–64^ Though all samples were treated identically after collection, protease inhibitors could not be added to the microdialysis vials during collection, which could have affected direct comparisons between compartments. However, CSF and bECF samples were continuously collected, prepared, and assayed under identical conditions by trained professionals; therefore, intersample variability should be negligible.

Elevated CSF levels of Tau proteins have been found in CSF^65^ as well as in bECF^15^ (for review, see a previous work^66^). We also found elevated Tau and p-Tau levels in both CSF and bECF samples. Tau and p-Tau levels in both biofluids showed a similar temporal pattern to S100B and NSE, but the rate of decrease over time appeared to be slower in the CSF. These changes can be interpreted in several ways, including a potentially long half-life of these axoskeletal proteins in the extracellular environment. The exact half-life of these protein biomarkers that are released from the intracellular environment is still not well known.^67^ Further, the extracellular environment can be altered by the severity and type of injury that can selectively activate extracellular proteases as part of the secondary injury process.^68–70^ In addition, Tau is an intrinsically disordered protein with a high propensity for self-aggregation,^71^ as also indicated by the presence of >200-kDa aggregates in our study.

### Limitations

This is a pilot study with limited numbers of patients who were all males. Technical issues include the use of standard CSF perfusion fluid that was used to perfuse the catheters, which may be suboptimal for protein recovery.^72^ Because of various issues, including patient safety, we were unable to collect sufficient quantities of bECF samples at all time points that would have enabled higher (e.g., daily or even higher) temporal resolution.

## Conclusion

Our pilot study focused on the acute stage of TBI in a clinically heterogeneous population. However, we found that the temporal pattern of changes in the CSF and bECF levels of four well-established neural injury markers were generally similar, suggesting that CSF levels of protein biomarkers can reflect intraparenchymal changes after TBI. The apparent multimeric S100B form detected in CSF and bECF samples may indicate other functions for S100B in the intracranial environment of the injured brain, for example, involvement in the inflammatory response after TBI upon binding to receptor RAGE.^42,43,73^ Given the biological significance of RAGE signaling in injury repair,^43^ these analyses need to be repeated on a larger scale using different analytical platforms. Our study is small, but it illustrates the value of both quantitative and qualitative analysis of protein biomarkers in serially sampled biofluids, especially CSF after TBI. It also demonstrates some of the challenges protein biomarker studies face vis-à-vis a complex, dynamically changing condition such as severe TBI.

## Supplementary Material

Supplementary Table S1

## Abbreviations Used

AIS: Abbreviated Injury Scale
AUC: area under the curve
bECF: brain extracellular fluid
cMD: cerebral microdialysis
CSF: cerebrospinal fluid
GCS: Glasgow Coma Scale
HRP: horseradish peroxidase
ISS: Injury Severity Score
NSE: neuron-specific enolase
p-Tau: phosphorylated Tau
RAGE: receptor for advanced glycation end product
S100B: S100 calcium-binding protein B
TBI: traumatic brain injury
WES: western analysis
