# Supplementary material for: Time-Dependent Changes in the Biofluid Levels of Neural Injury Markers in Severe Traumatic Brain Injury Patients–Cerebrospinal Fluid and Cerebral Microdialysates: A Longitudinal Prospective Pilot Study
Source: Neurotrauma Rep. 2023 Mar 1;4(1):107–17. doi: 10.1089/neur.2022.0076 (PMC9989523; doi:10.1089/neur.2022.0076)
Supplement: Supplementary Table S1 [file Supp_TableS1.docx]

**Supplementary Table 1** List of antibodies and dilutions used in the study

| **Markers** | **Manufacturer and Catalog #** | **Dilution** | ***Secondary antibody** |
| --- | --- | --- | --- |
| S100B | Abcam, ab41548 | 1:25 | Anti-Rabbit secondary |
| p-Tau | Cell Signaling, 11834 | 1:100 | Anti-Rabbit secondary |
| Tau | Cell Signaling, 4019 | 1:25 | Anti-Mouse Secondary |
| NSE | Abcam, ab53025 | 1:25 | Anti-Rabbit secondary |

*Primary antibody dilutions were optimized prior to the study ^32,62,74^. *Secondary antibodies were HRP-conjugated antibodies supplied by the manufacturer along with optimal dilutions ^32^.*
